# Impact of NVP Doping on the Holographic Properties of PQ/PMMA Holographic Storage Materials

**DOI:** 10.3390/polym17172321

**Published:** 2025-08-27

**Authors:** Lin Peng, Junhui Wu, Shujun Zheng, Hongjie Liu, Ruying Xiong, Xueyan Chen, Xu Zheng, Xiao Lin, Xiaodi Tan

**Affiliations:** 1 College of Photonic and Electronic Engineering, Fujian Normal University, Fuzhou 350117, China; pllsia@163.com (L.P.); junhuifjnu@foxmail.com (J.W.); qbx20240120@yjs.fjnu.edu.cn (S.Z.); qbx20220092@yjs.fjnu.edu.cn (H.L.); xiongruying0217@163.com (R.X.); 15980196902@163.com (X.C.); 15583687780@163.com (X.Z.); 2 Information Photonics Research Center, Key Laboratory of Optoelectronic Science and for Medicine of Ministry of Education, Fujian Provincial Key Laboratory of Photonics Technology, Fujian Provincial Engineering Technology Research Center of Photoelectric Sensing Application, Fujian Normal University, Fuzhou 350117, China

**Keywords:** holographic storage technology, photopolymer, comonomer, N-Vinyl-2-pyrrolidone, holographic properties

## Abstract

Photopolymer PQ/PMMA, as a pivotal material in the field of holographic storage, demonstrates significant application potential owing to its advantages, such as straightforward preparation processes, cost-effectiveness, and tunable thickness. However, its practical application is still constrained by the need for further enhancement in key performance indicators, including diffraction efficiency, photosensitivity, and anti-aging properties. In this study, N-vinylpyrrolidone (NVP) is employed as a comonomer. By precisely controlling the doping ratio, we systematically investigate the influence mechanism of different NVP doping concentrations on the holographic performance of NVP-PQ/PMMA materials. Research indicates that the introduction of NVP effectively increases the vinyl concentration in the PQ/PMMA matrix, thereby directly generating photoproducts with PQ during the photoreaction process and further enhancing the photopolymerization process. Consequently, the holographic performance of the novel NVP-PQ/PMMA material is improved in a multi-faceted manner compared to ordinary PQ/PMMA. Specifically, the diffraction efficiency is enhanced by 1.93 times, the photosensitivity is increased by 1.64 times, the material uniformity is improved by 38%, and the light-induced shrinkage rate is reduced by 39%. Additionally, NVP-PQ/PMMA materials exhibit excellent stability and aging resistance in high-temperature accelerated aging experiments. Doping with a monomer of specific structure enhances the optical properties, providing broad adaptability for further research on PQ/PMMA photopolymer materials.

## 1. Introduction

In the current era of big data, global data volume is growing exponentially, with structured and unstructured data projected to increase to 221.2 ZB by 2026 [1,2]. Traditional storage technologies such as hard disk drives and semiconductor flash memory devices face limitations in terms of cost-effectiveness, durability, and sustainability [1,3], making it difficult to meet the high-capacity, low-cost, and stable storage demands of massive data. In 1948, Dennis Gabor [4] first proposed the concept of holography. In recent years, holographic technology has found widespread applications in numerous fields, including three-dimensional imaging [5,6,7], information encryption and security [8,9,10], as well as holographic data storage [11,12]. Holographic storage technology, due to its unique multiplexing approach enabled by Bragg selectivity, is widely recognized as the next-generation data storage technology [13,14,15]. It is capable of storing data in three-dimensional space, offering a large storage capacity, and featuring simultaneous read-and-write capabilities, thereby enabling high-speed data transmission [10,16].

Research on holographic storage media mainly focuses on photorefractive materials [17,18,19,20,21], photochromic material [22,23,24,25,26] and photopolymer materials [27,28,29]. Among a multitude of holographic storage materials, PQ/PMMA photopolymers are extensively employed for holographic data storage due to their high resolution, real-time recording capability, low cost [30,31], and polarization sensitivity [32,33]. However, there remains room for improvement in aspects such as diffraction efficiency, photosensitivity, and anti-aging properties [34]. To enhance the performance of PQ/PMMA, researchers have proposed various modification strategies, such as co-doping with other monomers to increase the crosslinking density, doping with nanoparticles [35] like graphene [36,37], and adding plasticizers. Additionally, attempts have been made to improve the capability of reconstructing holograms using polarization-sensitive materials [38,39]. However, these methods often struggle to balance multiple performance metrics simultaneously, highlighting an urgent need for a modification strategy capable of systematically elevating the overall performance of PQ/PMMA.

The regulation of C=C bond concentration in the PQ/PMMA photopolymerization system is a critical factor for optimizing material properties [40,41]. The underlying mechanism lies in the fact that the radicals generated from the photosensitizer PQ upon photoexcitation can efficiently react with the C=C bonds in the system, thereby promoting the formation of photoproducts. In this study, N-vinyl pyrrolidone (NVP) was selected as a functional modifying monomer, whose molecular structure exhibits the following characteristics: (1) a highly reactive C=C double bond; (2) a carbonyl polar group; (3) a five-membered heterocyclic structure containing an N atom. These structural features give NVP multiple functional advantages, allowing the formation of high-performance polymer materials through copolymerization [42,43,44]. From the perspective of molecular structure-property relationship, the cyclic structure of NVP can significantly enhance the rigidity of the polymer [45], and effectively suppress photo-induced volume shrinkage by regulating the density of the cross-linked network. Kinetic studies have demonstrated that its high vinyl concentration can significantly improve the optical properties of the material [40,41]. Thermodynamic analysis reveals that the carbonyl structure of NVP not only enhances the cohesive strength and thermal resistance of the polymer [46], increases the material’s aging resistance, but also exerts a certain degree of polymerization inhibition during the thermal polymerization stage of the PQ/PMMA system [47]. This results in a higher residual amount of MMA molecules in the material system doped with NVP under the same conditions of stirring pre-polymerization and baking thermal polymerization. In terms of solubility characteristics, NVP, as an excellent organic solvent, can effectively enhance the solubility of PQ in the PMMA matrix, thereby optimizing the effective concentration distribution of the photosensitizer. Furthermore, its outstanding thermal stability [45] and broad application potential (such as in UV cross-linking and temperature-sensitive hydrogels) make it an ideal modifier.

Owing to the multifunctional advantages of NVP, this study incorporates NVP as a comonomer into the PQ/PMMA system to fabricate a novel NVP-PQ/PMMA photopolymer material and explore its holographic storage performance, including parameters such as diffraction efficiency, photosensitivity, uniformity, and aging resistance. Given the potential nonlinear relationship between different N-vinyl-2-pyrrolidone (NVP) doping concentrations and material performance, as well as the need to balance the enhancing effects of NVP with the possible adverse impacts of excessive doping, this research employs a gradient concentration design (0.00 wt%, 0.05 wt%, 0.10 wt%, 0.15 wt%, and 0.20 wt%). By employing this method, the relationship between N-vinyl pyrrolidone (NVP) concentration and optical properties can be established, enabling a systematic investigation into the influence mechanism of different NVP doping concentrations on material performance. In this experiment, Gel Permeation Chromatography (GPC) was utilized to test the molecular weight changes, and Thermogravimetric Analysis (TGA) was conducted to analyze the variations in material mass with temperature during the photoreaction process. Furthermore, a dual-beam interference holographic testing system was employed to evaluate parameters such as diffraction efficiency, material uniformity, photosensitivity, refractive index modulation depth, and photoinduced shrinkage. During this testing process, when exposed to green laser light, the PQ/PMMA material undergoes interference between the signal light and the reference light, resulting in the formation of periodic bright and dark fringes. In the bright regions, photopolymerization reactions occur, wherein the photosensitizer PQ, upon excitation, reacts with MMA and PMMA to generate photoproducts, while essentially no reaction takes place in the dark regions. Due to the effect of concentration gradients, PQ and MMA in the dark regions diffuse toward the bright regions, whereas the macromolecular photoproducts in the bright regions find it difficult to diffuse back in the opposite direction owing to steric hindrance. This leads to the establishment of a pronounced concentration difference in chemical components between the bright and dark regions. Such a concentration gradient ultimately gives rise to a holographic grating with refractive index modulation. Additionally, accelerated aging experiments are conducted to evaluate the material’s aging resistance.

The research results indicate that the introduction of NVP significantly improves the holographic performance of PQ/PMMA. When the NVP doping concentration is 0.20 wt%, the diffraction efficiency increases to 19.5% (an improvement of 1.93 times), and the photosensitivity increases by 1.64 times, reaching 1.18 cm J^−1^. When the NVP doping concentration is 0.10 wt%, the material uniformity improves by 38%, and the volume shrinkage rate decreases to 0.24% (compared to 0.38% in the control group). Moreover, the addition of NVP effectively delays the material’s light-induced decay and enhances its aging resistance. This study establishes a quantitative relationship between NVP concentration and material properties, providing a crucial theoretical foundation for optimizing the performance of holographic storage materials. Based on the obtained data, subsequent research can select the optimal doping concentration for different performance indicators according to practical application requirements. This holds significant guiding importance for advancing the practical application of holographic storage materials.

## 2. Materials and Methods

**Figure 1:**
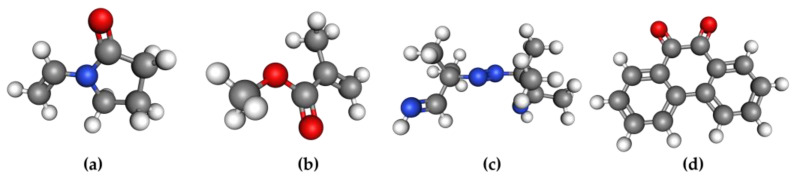
Red: O atom; gray: C atom; white: H atom; blue: N atom. (**a**) N-vinyl-2-pyrrolidinone (NVP), (**b**) Methyl methacrylate (MMA), (**c**) 2,2-azobis (2-methylpropionitrile) (AIBN), (**d**) Phenanthraquinone (PQ).

**Figure 2:**
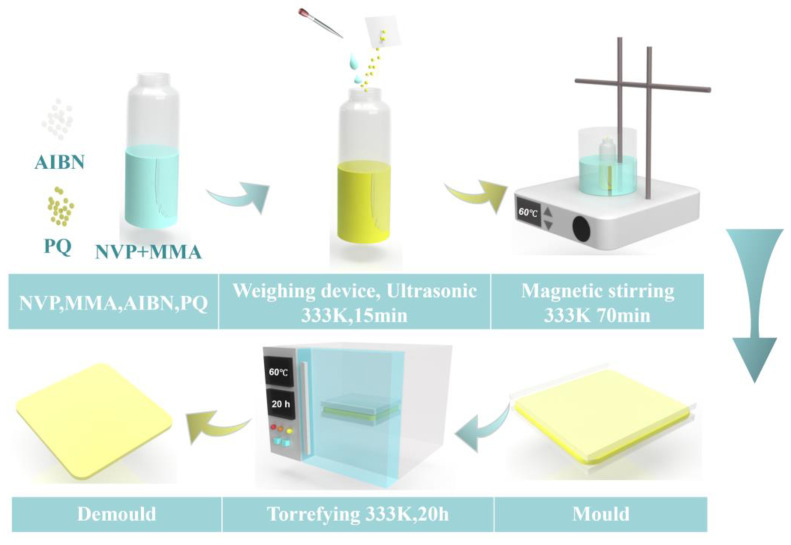
Preparation process steps.

Materials. The monomer N-vinyl-2-pyrrolidone (NVP, purity: 99%, containing 100 ppm NaOH stabilizer) was purchased from Saen Chemical Technology (Shanghai) Co., Ltd., Shanghai, China. Monomers methyl methacrylate (MMA, purity: 99.5%), thermal initiator 2,2-azobis(2-methylpropionitrile) (AIBN, purity: 99%), photosensitizer phenanthrenequinone (PQ, purity: 99%), and tetrahydrofuran (THF, purity: ≥99.5%) were purchased from Shanghai Macklin Biochemical Technology Co., Ltd., Shanghai, China. NVP-PQ/PMMA materials with different doping concentrations were prepared using MMA and NVP as monomers, AIBN as the thermal initiator, and PQ as the photosensitizer. Their chemical structures are shown in Figure 1:

Sample preparation. The material preparation process is illustrated in Figure 2. This study prepared PQ/PMMA photopolymer materials with different NVP doping concentrations. The specific experimental procedure is as follows: MMA and NVP were precisely mixed in a 20 g sample bottle at specific ratios to systematically configure (NVP + MMA) composite systems with NVP mass fractions of 0.00 wt%, 0.05 wt%, 0.10 wt%, 0.15 wt%, and 0.20 wt%. This allowed for an in-depth study of the impact of NVP doping on the holographic properties of PQ/PMMA photopolymer materials. During the experiment, the reaction system component ratios were strictly controlled, maintaining MMA/AIBN/PQ = 100:1:1, and 1.0 wt% PQ and 1.0 wt% AIBN were sequentially added. The proportions of each component in the MMA mass ratio are shown in Table 1.

To ensure thorough mixing of all components, the mixture was placed in a 333 K constant-temperature water bath and subjected to ultrasonic treatment for 15 min. This experimental protocol was optimized based on references [31,46,48]. Subsequently, the vials were transferred to a magnetic stirrer and continuously stirred at a stirring rate of 600 rpm under a constant temperature of 333 K for 70 min until the solution attained a viscous state. After the stirring was halted, the prepolymer was injected into a specially designed mold with a thickness of 0.5 mm and then placed in a constant-temperature oven at 333 K for 20 h to complete the polymerization reaction. The cured samples were then subjected to a 15-min freezing treatment at a low temperature of −18 °C to facilitate demolding, ultimately yielding NVP-PQ/PMMA sheet-like holographic photopolymer materials with a thickness of 0.5 mm. All prepared samples were packaged and stored in aluminum foil bags to shield them from light.

**Figure 3:**
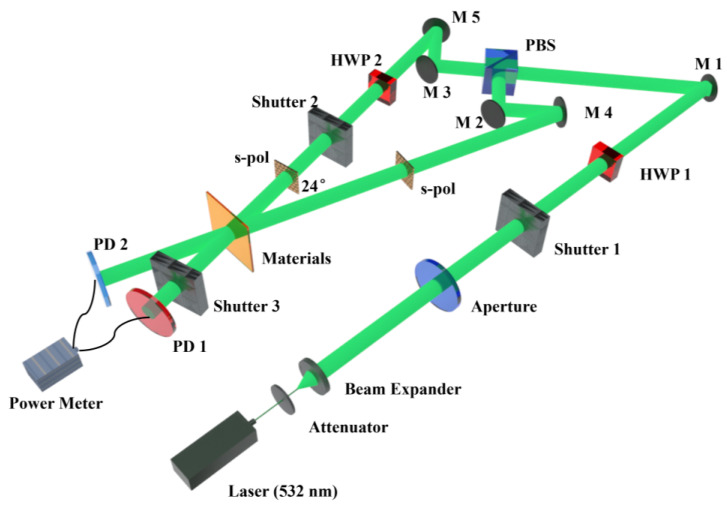
Optical path for measuring the diffraction efficiency of materials (HWP: half-wave plate; PBS: polarized light splitter; M: plane mirror; PD: Photodetector).

Holographic Recordings and Measurements. The experimental optical setup employed for measuring holographic performance is depicted in Figure 3. The all-solid-state single-longitudinal-mode green laser (MSL-FN-532-300 mW) utilized in the experiments was purchased from Changchun New Industries Optoelectronics Technology Co., Ltd, Jilin, China. Key optical components, such as the half-wave plate (HWP) and diaphragm, were sourced from Daheng New Epoch Technology, Inc, Beijin, China. The photodetector (PD) was acquired from Thorlabs, Inc, Shanghai, China.

The detailed description of the optical path for holographic diffraction testing is as follows: The 532 nm green laser light source initially passes through an attenuator to regulate the light intensity. After beam expansion, it proceeds through a half-wave plate (HWP1) and is subsequently split into two beams by a polarization beam splitter (PBS). The polarization state of the signal beam is set to s-polarization (s-pol), while the polarization state of the reference beam is also adjusted to s-pol using another half-wave plate (HWP2). In this experiment, conventional holographic recording is employed, with a single-beam power of 25 mW, a beam diameter of 5 mm, and an interference angle of 24° [49]. The grating recording and reconstruction processes are controlled by shutters. Specifically, for interference recording, shutter 1 and shutter 2 are opened while shutter 3 is closed, maintaining this state for 6 s to record the interference pattern onto the material. For diffraction light readout, shutter 1 is opened, shutter 2 is closed, and shutter 3 is opened, holding this configuration for 0.6 s to read the diffracted light from the material. This recording and readout cycle is repeated iteratively until the intensity of the diffracted light no longer increases.

Diffraction efficiency serves as a core metric for evaluating the rate and scale of grating formation in a material during exposure, and it is of paramount importance in assessing the material’s potential to become a high-quality holographic storage medium. The diffraction efficiency (*η*) is expressed as follows:

(1) $$\;\eta =\frac{I_{1}}{I_{1}+I_{0}}$$

where *I*_0_ and *I*_1_ represent the transmitted light intensity and the first-order diffraction light intensity, respectively.

Photosensitivity is a parameter that describes the rate of grating formation and serves as a crucial factor influencing the recording performance of the medium. To meet the high-speed storage requirements of holographic storage systems, the storage medium must possess sufficient photosensitivity. The photosensitivity coefficient (*S*) of the material is calculated as follows [50]:

(2) $$\;S=\frac{1}{Id}(\frac{\partial \sqrt{\eta }}{\partial t})$$

where *I* is the intensity of the recording wave (0.102 W/cm^2^), *d* is the material thickness (0.5 mm), and *η* is the diffraction efficiency.

According to Kogelnik’s coupled-wave theory [51], the refractive index modulation (Δ*n*) of the photopolymer is defined as:

(3) $$\;∆n\left(t\right)=\frac{\lambda \cos\theta_{0}}{\pi d}\sin^{-1}\sqrt{\eta }$$

where *λ* is the recording information wavelength (532 nm), *θ*_0_ is the Bragg angle between the signal beam and the reference beam, and *d* is the material thickness (0.5 mm).

Generally speaking, due to the excessively rapid response time during holographic data recording, photopolymers often experience significant photo-induced shrinkage. This issue readily leads to severe grating distortions and Bragg detuning phenomena, which in turn can cause errors in the holographic data retrieval process. The volume shrinkage ratio (*σ*) [52] of the sample is defined as:

(4) $$\;\sigma =1-\frac{\tan\theta_{theo}}{\tan\theta_{exp}}$$

where *θ_theo_* and *θ_exp_* are the theoretical and experimental Bragg angles of the photopolymer after double-beam interference, respectively.

During the testing of the collinear holographic storage system, it is necessary to evaluate the quality of the retrieved reconstructed images to validate the effectiveness of the proposed scheme. In this paper, the Bit Error Rate (*BER*) and Signal-to-Noise Ratio (*SNR*) [53] are selected as evaluation metrics, and their respective mathematical formulations are presented as follows:

(5) $$\;BER=\frac{N_{error}}{N_{total}}$$

(6) $$SNR=\frac{\mu_{ON}-\mu_{OFF}}{{{(\sigma }_{ON}^{2}+\sigma_{OFF}^{2})}^{\frac{1}{2}}}$$

In Equation (5), *N_total_* denotes the total number of data symbols l in the original information light pattern, whereas *N_error_* represents the number of erroneous data symbols in the reconstructed image. In Equation (6), *μ_ON_* and *μ_OFF_* respectively denote the mean values of the *ON* and *OFF* pixels, while *σ_ON_* and *σ_OFF_* represent the variances of the *ON* and *OFF* pixels.

Characterization. Firstly, a UV-5200PC ultraviolet-visible spectrophotometer [54] was used to measure NVP-PQ/PMMA materials with different doping concentrations, investigating whether the introduction of the comonomer NVP affects the optical absorption characteristics of the PQ/PMMA material. The absorbance (A) was calculated using Equation (7). Secondly, Fourier-transform infrared spectroscopy (FT-IR) spectra of NVP-PQ/PMMA materials with different doping concentrations before and after exposure were measured to analyze the role of NVP during the thermal and photoreaction stages. Subsequently, a Shimadzu CTO-20A gel permeation chromatography system was used to measure the polymer molecular weight, with an elution temperature of 313 K, an elution rate of 1 mL min^−1^, and tetrahydrofuran (THF, 99.5%) as the mobile phase. Finally, a TGA/DSC1/1100LF thermogravimetric analyzer was used to investigate the mass change of samples with temperature, with a temperature range set at 30~600 °C, a heating rate of 10 °C min^−1^, and experiments conducted under a nitrogen (N_2_) environment.

(7) $$\;A=-\ln\frac{I_{t}}{I_{0}}$$

where *I*_0_ and *I_t_* are the intensities of the incident and transmitted light, respectively.

## 3. Results and Discussion

### 3.1. Holographic Recording Performance of Different Concentrations of NVP-PQ/PMMA

**Figure 4:**
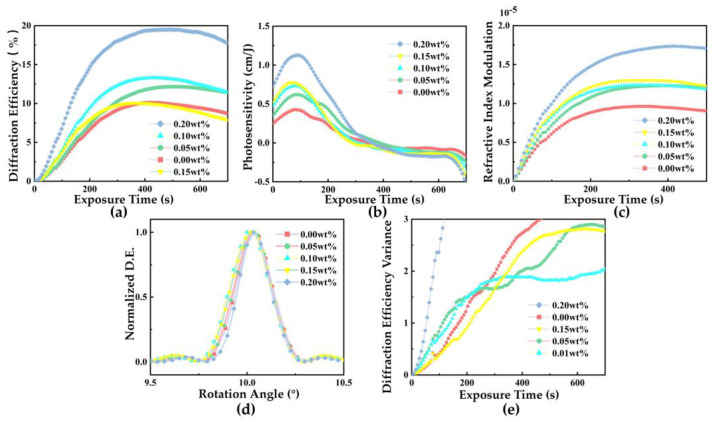
(**a**) Exposure time-dependent diffraction efficiency of different concentrations of NVP−PQ/PMMA, (**b**) Exposure time-dependent photosensitivity of different concentrations of NVP−PQ/PMMA, (**c**) Exposure time-dependent refractive index modulation Δ*n* of different concentrations of NVP−PQ/PMMA, (**d**) Rotation angle (°) dependent Normalized D.E. of different concentrations of NVP−PQ/PMMA, and (**e**) Exposure time-dependent diffraction efficiency variance of different concentrations of NVP−PQ/PMMA.

Leveraging the multifaceted functional advantages of NVP within the polymer system, this study systematically investigated the impact mechanism of varying NVP doping concentrations on the performance of PQ/PMMA holographic storage materials. By measuring key parameters such as diffraction efficiency, photosensitivity, refractive index modulation, photo-induced shrinkage, and uniformity, the experimental results are illustrated in Figure 4, establishing a quantitative relationship between NVP concentration and material performance.

Figure 4 illustrates the systematic influence of varying NVP doping concentrations on the performance of NVP-PQ/PMMA holographic storage materials. As shown in Figure 4a, with the increase in NVP doping concentration, the diffraction efficiency of the material exhibits a significant upward trend. When the doping concentration reaches 0.20 wt%, the diffraction efficiency increases from 10.1% to 19.5%, representing an enhancement by a factor of 1.93.

In terms of photosensitivity (Figure 4b), the sample doped with 0.20 wt% NVP achieves an outstanding performance of 1.18 cm·J^−1^, marking a 1.74-fold improvement compared to the undoped sample (0.43 cm·J^−1^) at 0.00 wt%.

The refractive index modulation (Δ*n*) results, calculated based on coupled-wave theory (Figure 4c), reveal that the Δ*n* value for the 0.00 wt% sample is 0.9 × 10^−5^, whereas the Δ*n* value for the 0.20 wt% doped sample significantly increases to 1.7 × 10^−5^. This breakthrough holds substantial significance for PQ/PMMA holographic polymers.

The photo-induced shrinkage deviation angles for different doping concentrations are depicted in Figure 4d. The NVP-PQ/PMMA sample with 0.10 wt% NVP exhibits minimal positional drift (approximately 0.024°) in its in situ volume shrinkage characteristics, which is lower than that of the 0.00 wt% PQ/PMMA sample (approximately 0.038°). According to the volume shrinkage rate Formula (4), the volume shrinkage rates for the 0.00 wt% and 0.10 wt% doped materials are calculated to be 0.38% and 0.24%, respectively. Thus, the photo-induced shrinkage effect is reduced by 37%. This can be attributed to two factors: firstly, the residual monomers after thermal polymerization are more uniformly dispersed within the PMMA matrix; secondly, the time required for the material to reach its maximum diffraction efficiency is shorter, indicating a significant reduction in photopolymerization time.

Finally, regarding uniformity, in addition to the aforementioned excellent holographic properties, the experimental results also reveal that the incorporation of NVP monomers can improve the non-uniform characteristics of the original PQ/PMMA material. In this experiment, the diffraction efficiency at multiple points on the same NVP-PQ/PMMA material sheet with different doping concentrations was tested (Figure S1), and the variance in diffraction efficiency was calculated to assess material uniformity. The results, shown in Figure 4e, indicate that when the NVP doping concentration is 0.10 wt%, the variance in diffraction efficiency is 1.84, whereas the variance for the 0.00 wt% PQ/PMMA is 2.97. Thus, the material uniformity is enhanced by 38%. These results fully demonstrate the significant effect of NVP doping in improving the comprehensive performance of PQ/PMMA holographic storage materials.

### 3.2. Microphysical Mechanism of NVP-PQ/PMMA Samples

**Figure 5:**
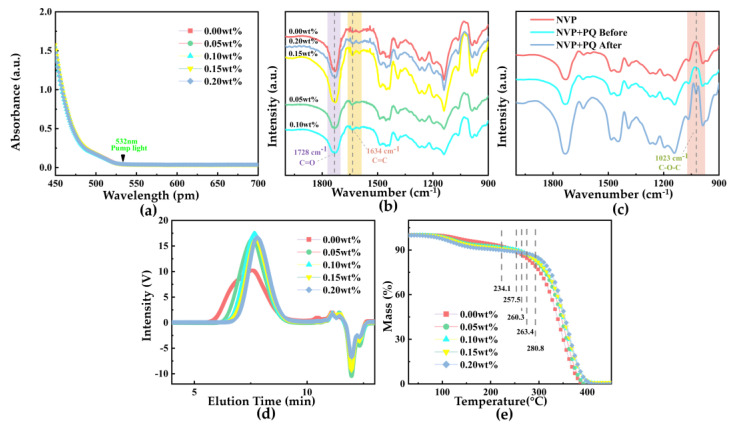
(**a**) Absorption spectra of different concentrations of NVP−PQ/PMMA, (**b**) FT−IR spectra of different concentrations of NVP−PQ/PMMA, (**c**) FT−IR of NVP and NVP + PQ Before and After Exposure, (**d**) GPC traces of different concentrations of NVP−PQ/PMMA, and (**e**) TGA results of different concentrations of NVP−PMMA.

To verify the role of NVP monomer during the thermal polymerization reaction and understand the microscopic mechanism by which NVP enhances the holographic performance of PQ/PMMA photopolymers, various characterization experiments were conducted on NVP-PQ/PMMA materials with different doping concentrations. The results are shown in Figure 5.

The UV-Vis absorption spectra of NVP-PQ/PMMA materials were measured, and the results are shown in Figure 5a. The absorption coefficient curves for samples with different doping concentrations remain consistent overall, indicating that the introduction of NVP monomer does not significantly affect the raw material’s light absorption. The sample exhibits only minimal absorption in the 532 nm wavelength band. Therefore, to prevent excessive light absorption from affecting the material, a 532 nm green laser was selected for subsequent experiments.

Further measurements of Fourier Transform Infrared Spectroscopy (FT-IR) spectra were conducted on the materials with varying NVP concentrations, and the results are shown in Figure 5b. The absorption peak near 1728 cm^−1^ corresponds to the stretching vibration of the C=O bond, while the characteristic peak at 1634 cm^−1^ is attributed to the stretching vibration of the C=C double bond, both of which are presumably derived from the PQ/PMMA substrate. Compared to the material with 0.00 wt% NVP, the introduction of NVP at different doping concentrations did not result in the emergence of new peaks or significant changes in the characteristic transmission spectra. This indicates that the NVP monomer did not react with the PMMA chains during the thermal reaction stage, thereby providing a basis for subsequent reactions with the photosensitizer PQ to generate photoproducts during the photoreaction stage. To investigate the actual mechanism of NVP, FT-IR experiments were performed on samples of NVP, NVP+PQ before exposure, and NVP + PQ after exposure. The experimental results, as shown in Figure 5c, reveal the emergence of a new peak at 1023 cm^−1^ in the NVP+PQ sample after exposure, which is identified as the C-O-C bond. This suggests that the vinyl group (C=C bond) in the NVP monomer can react with the carbonyl group (C=O bond) in the photosensitizer PQ to produce photoproducts containing C-O-C bonds.

The Gel Permeation Chromatography (GPC) elution curves of NVP-PQ/PMMA with varying doping concentrations of NVP were tested, and the results are shown in Figure 5d. According to the working principle of gel permeation chromatography, polymers with larger molecular weights have faster flow rates and are detected earlier, appearing at the front positions on the GPC curve’s time coordinate. Therefore, according to Figure 5d, with increasing material doping concentration, the molecular volume of PMMA polymers gradually decreases. The molecular weights are shown in Table 2.

As the doping concentration of NVP (N-vinyl-2-pyrrolidone) increases, the number-average molecular weight (Mn) of the NVP-PQ/PMMA pre-polymerization solution decreases from 1.19 × 10^5^ g mol^−1^ to 0.72 × 10^5^ g mol^−1^, while the weight-average molecular weight (Mw) drops from 6.70 × 10^5^ g mol^−1^ to 1.56 × 10^5^ g mol^−1^. This phenomenon indicates that no significant chemical reaction occurs between NVP monomers and PMMA long-chain polymers during the thermal polymerization process. The reduction in molecular weight confirms that NVP exhibits a significant polymerization inhibition effect during thermal polymerization. Under the same reaction conditions, the decrease in the molecular weight of PMMA polymers leads to a higher residual concentration of MMA (methyl methacrylate) monomers in the system. Given that the binding energy of PQ/MMA is lower than that of PQ/PMMA, more PQ/MMA photoproducts are generated more rapidly in the subsequent photoreaction stage. This process significantly enhances the system’s response speed and improves the photosensitivity in holographic performance. Additionally, the accelerated molecular binding process promotes an increase in molecular diffusion rate [55,56,57], thereby improving diffraction efficiency to a certain extent.

The aforementioned phenomena can be attributed to the steric hindrance effect of the pyrrolidone ring in NVP molecules. As the proportion of NVP increases, its steric hindrance effect becomes more pronounced, resulting in a shortening of the copolymer molecular chain length. Specifically, the steric hindrance of the pyrrolidone ring restricts the continuous copolymerization of the four monomers. When the molecular chain length reaches a critical value, the polymerization reaction terminates [58]. Meanwhile, the polymer dispersity index (PDI) serves as a crucial metric for evaluating the uniformity of particle size distribution. In Table 2, the PDI decreases from 5.61 to 2.18, indicating that as the doping concentration increases, the mass-volume distribution of PMMA long-chain polymers within the system becomes more uniform. This result aligns with the findings from previous studies, which suggest that NVP (N-vinyl pyrrolidone) monomers enhance material uniformity.

Simultaneously, thermogravimetric analysis (TGA) experiments were used to further verify the microscopic reaction mechanism of NVP-PQ/PMMA with different doping concentrations. From the TGA curves in Figure 5e, as the NVP concentration increases from 0.00 wt% to 0.20 wt%, the initial degradation temperature of the first stage decreases from 111.6 °C to 76.6 °C. This stage primarily involves the volatilization of MMA small molecules. From Table 3, the percentage of residual MMA molecules in the samples can be analyzed as 5.93%, 7.42%, 8.08%, 9.57%, and 10.68%, respectively, indicating that NVP can regulate the residual MMA content in PMMA. The initial degradation temperature of the second stage gradually increases from 234.1 °C to 280.8 °C, primarily involving the thermal decomposition of PMMA macromolecular polymers. This suggests that the introduction of NVP monomer makes PMMA macromolecular polymers less prone to decomposition, significantly increasing thermal stability and thus enhancing anti-aging properties.

The DTG results shown in Figure S2 clearly indicate that the maximum degradation rate temperature corresponding to the second stage slightly increases from 350.8 °C to 356.6 °C, proving that NVP does not undergo grafting reactions with PMMA. The introduction of NVP increases the residual vinyl content in the PMMA matrix after thermal polymerization. Previous studies have shown that under illumination, the photosensitizer PQ can react with C=C double bonds, thereby enhancing the reaction rate, which is one of the reasons for the significant improvement in photosensitivity of photopolymers.

### 3.3. Collinear Holographic Information Recording of Different Concentrations of NVP−PMMA/PQ

**Figure 6:**
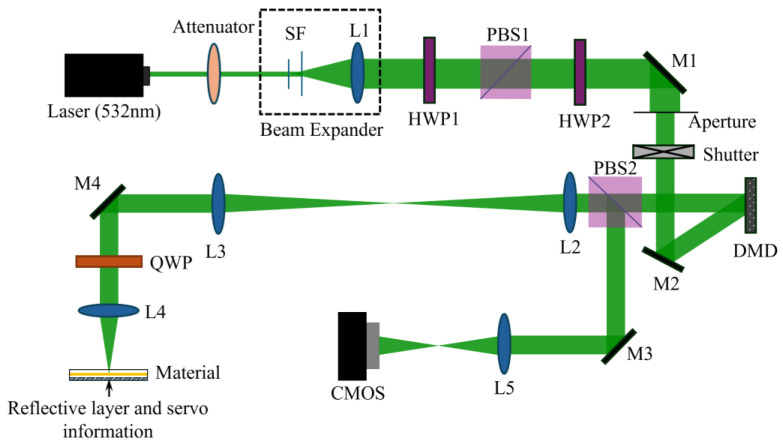
Schematic of the collinear holographic storage system (HWP: half-wave plate; QWP: quarter wave plate; M: mirror; PBS: polarization beam splitter; SF: Spatial Filter; DMD: Digital Micromirror Device; CMOS: image sensor).

The collinear holographic data storage system, recognized as a holographic data storage solution with enhanced compactness and compatibility, operates on the principle of splitting a single light beam into two components: the information beam and the reference beam. These two beams are then brought together to interfere, enabling the storage of information. The optical path diagram is illustrated in Figure 6. During the recording process, data is first encoded, and the encoded data page is subsequently loaded onto the optical path using a Digital Micromirror Device (DMD). The data page converges near the focal plane of the objective lens to facilitate information recording. In the retrieval phase, merely loading the external original reference beam onto the DMD suffices to reconstruct the image information carried by the signal beam.

**Figure 7:**
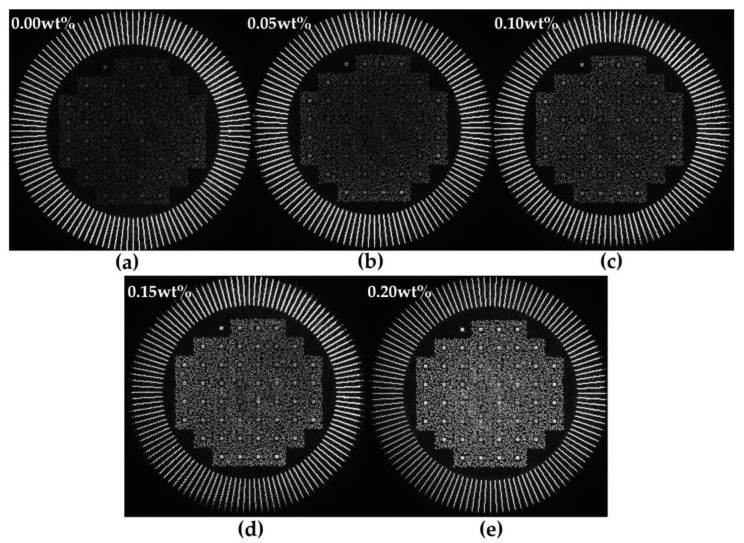
The read−out images of (**a**) 0.00 wt% NVP−PQ/PMMA, (**b**) 0.05 wt% NVP−PQ/PMMA, (**c**) 0.10 wt% NVP−PQ/PMMA, (**d**) 0.15 wt% NVP−PQ/PMMA and (**e**) 0.20 wt% NVP−PQ/PMMA after exposure for 150 s using a collinear beam.

We fabricated NVP-PQ/PMMA yellow thin-sheet materials (50 mm × 50 mm × 0.5 mm) with reflective mirrors, featuring different doping concentrations. These materials were tested on a collinear holographic data storage system by employing a method of multiple recordings and readings at 5-s intervals. As depicted in Figure 7, the experimental results for NVP-PQ/PMMA with varying doping concentrations are shown, where the outer ring represents the reference beam and the central portion displays the reconstructed data page. The data page consists of a synchronization marker in the top-left corner and 51 data sub-pages. Each sub-page contains 32 data symbols and a sub-page synchronization marker. All the symbols within the data page were sequentially extracted and compared with the ground-truth image; symbols that matched were considered correct, while mismatches were deemed incorrect.

**Figure 8:**
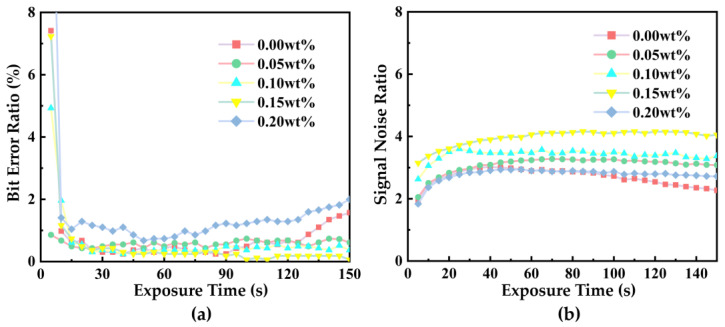
Collinear holographic storage system images: the recording time-dependent BER (**a**) and SNR (**b**) results of different concentrations of NVP−PQ/PMMA.

The bit error rate (BER) and signal-to-noise ratio (SNR) were calculated using Equations (5) and (6) to evaluate the quality of the reconstructed image, with the results shown in Figure 8. As can be seen from Figure 8a, the 0.00 wt% NVP-PQ/PMMA material maintains a relatively low bit error rate during exposure times of less than 100 s, after which the bit error rate begins to exhibit an upward trend. Samples with NVP concentrations of 0.05, 0.10, and 0.15 wt% exhibited consistently low and stable BER values during prolonged recording and readout processes. Among these, the 0.15 wt% sample demonstrated the lowest BER, approximately 0.06% (a 76% reduction compared to the lowest BER of 0.25% observed for the 0.00 wt% sample). In contrast, the 0.20 wt% sample displayed the highest and most unstable BER among all doping concentrations tested. As can be seen from Figure 8b, when the doping concentration is below 0.15 wt%, the signal-to-noise ratio (SNR) of the sample increases with the rising concentration. The material with a concentration of 0.15 wt% exhibits the highest SNR, approximately 4.16 (representing a 38% improvement compared to the SNR of 3.02 at 0.00 wt%). When the concentration further increases to 0.20 wt%, the SNR decreases significantly. This indicates that when the NVP concentration reaches 0.20 wt%, it is detrimental to recording information in a collinear holographic storage system. This phenomenon stems from the nonlinear effect of the NVP concentration. When the NVP concentration is 0.15 wt%, its role as a comonomer achieves an optimal balance, promoting the uniformity of the polymer network, enhancing carrier transport, and reducing moderate cross-linking defect states. However, when the concentration rises to 0.20 wt%, an excess of NVP may lead to incomplete local polymerization, thereby affecting the holographic image storage performance. In summary, the NVP-PQ/PMMA material with an NVP concentration of 0.15 wt% demonstrates both a low bit error rate and a high SNR in a coaxial holographic storage system, yielding the best overall performance.

### 3.4. Aging Life of Different Concentrations of NVP−PMMA/PQ

Transmissive NVP-PQ/PMMA samples with varying doping concentrations were individually placed in an intensity holographic diffraction characteristic testing setup (Figure 3). Grating recording and reading were performed on nine distinct regions of each sample, as illustrated in Figure S3. The recording process was terminated when the diffraction intensity first reached its peak value.

**Figure 9:**
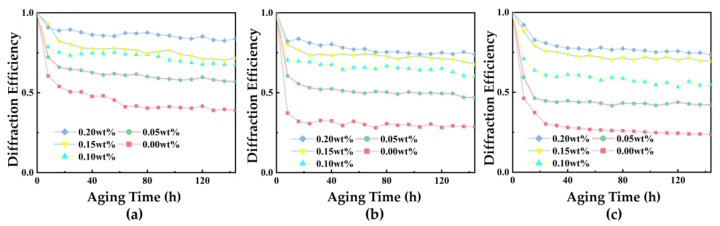
Normalized diffraction efficiency of NVP−PQ/PMMA at different concentrations under aging at (**a**) 70 °C, (**b**) 75 °C, and (**c**) 78 °C.

Subsequently, the samples were placed in ovens at 70 °C, 75 °C, and 78 °C for accelerated aging under harsh environmental conditions. Samples were taken out at equal intervals (8 h) for intensity holographic diffraction characteristic testing, and the changes in grating diffraction intensity values were recorded and normalized, with the results shown in Figure 9.

During aging at different temperatures, a consistent pattern was observed: as the doping concentration of NVP-PQ/PMMA increased, the attenuation of diffraction intensity gradually decreased. Furthermore, a higher aging temperature resulted in a greater attenuation of diffraction intensity for materials with the same doping concentration. Additionally, as the aging time elapsed, the rate of decrease in grating intensity within the material gradually slowed down. After 144 h of aging at an aging temperature of 70 °C, the diffraction intensity of the 0.20 wt% NVP-PQ/PMMA sample decreased to 83.6%, representing a 44.7% increase compared to the 38.9% diffraction intensity of the 0.00 wt% material. At an aging temperature of 75 °C, the diffraction intensity of the 0.20 wt% NVP-PQ/PMMA sample declined to 71%, which was a 47.3% enhancement over the 23.7% diffraction intensity of the 0.00 wt% material. At an aging temperature of 78 °C, the diffraction intensity of the 0.20 wt% NVP-PQ/PMMA sample dropped to 63.5%, showing a 34.8% improvement compared to the 28.7% diffraction intensity of the 0.00 wt% material. This is because NVP, a functional monomer with a carbonyl polar group and an N heterocycle, can enhance the material’s cohesive strength, heat resistance, and adhesive surface bonding strength. As the NVP mass fraction increases, the rigidity of the polymer gradually enhances, simultaneously increasing the material’s cohesive strength and avoiding carbonization or decomposition at high temperatures. Thus, introducing NVP monomer into the PQ/PMMA system enhances anti-aging properties, with 0.20 wt% NVP-PQ/PMMA exhibiting the strongest anti-aging properties. This is also consistent with the TGA experimental results. As the NVP concentration increases, the second-stage degradation temperature rises, making PMMA macromolecular polymers less prone to decomposition and thus enhancing anti-aging properties.

## 4. Conclusions

In this study, N-vinyl pyrrolidone (NVP) monomers were incorporated into the PQ/PMMA photopolymer system, leading to the successful fabrication of NVP-PQ/PMMA photopolymer materials with varying NVP doping concentrations. The experimental findings reveal that the introduction of NVP monomers into the PQ/PMMA system can substantially enhance the holographic properties of the materials. Specifically, the 0.10 wt% NVP-PQ/PMMA material significantly improves the material’s resistance to photoinduced shrinkage and enhances its uniformity. The 0.15 wt% NVP-PQ/PMMA sample demonstrates both a low bit error rate (BER) and a high signal-to-noise ratio (SNR) in a collinear holographic storage system, ensuring the reliability of information recording and retrieval. When the NVP doping concentration is 0.20 wt%, the material exhibits high diffraction efficiency, excellent photosensitivity, notable refractive index modulation, and favorable anti-aging stability.

In practical applications, the optimal NVP doping concentration can be selected based on specific holographic performance requirements. Furthermore, this study employs multiple microscopic characterization techniques to thoroughly investigate the microscopic mechanisms underlying the enhanced holographic performance due to NVP modification. The improvement in optical properties brought about by dopant monomers with specific structures provides a theoretical basis for the further functional design of PQ/PMMA photopolymer materials and expands their potential applications in high-speed holographic data storage.

## Figures and Tables

**Table 1:** Concentration ratio of each component in the prepared sample.

Concentration of NVP (wt%)	NVP (g)	MMA (g)	AIBN (g)	PQ (g)
0.00	0.00	20	0.2	0.2
0.05	0.01			
0.10	0.02			
0.15	0.03			
0.20	0.04			

**Table 2:** Mn, Mw and PDI of Different Concentrations of NVP-PQ/PMMA.

Samples (wt %)	Mn (g/mol)	Mw (g/mol)	PDI
0.00	119,258	669,613	5.61
0.05	108,554	295,973	2.73
0.10	91,523	218,215	2.83
0.15	81,279	182,657	2.24
0.20	71,567	155,764	2.18

**Table 3:** TGA Data of Different Concentrations of NVP−PQ/PMMA Materials.

Sample (wt %)	Ta1 (°C)	Tb1 (°C)	MMA (%)	Ta2 (°C)	Tb2 (°C)	M2 (%)
0.00	111.6	135	5.93	234.1	350.8	91.4
0.05	94.1	123.3	7.42	257.5	350.8	89.7
0.10	88.3	117.5	8.08	260.3	356.6	89.2
0.15	82.5	111.6	9.57	263.4	356.6	88.5
0.20	76.6	111.6	10.68	280.8	356.6	87.2

Ta: Initial temperature of degradation. Tb: Temperature at the maximum degradation rate. M: Weight loss percent during the degradation stage.

## Data Availability

Data are contained within the article and Supplementary Materials.

## Supplementary Materials

The following supporting information can be downloaded at: https://www.mdpi.com/article/10.3390/polym17172321/s1, Figure S1: The diffraction efficiencies at multiple points on the same NVP-PQ/PMMA films with different doping concentrations, where (a) is 0.00 wt% NVP-PQ/PMMA, (b) is 0.05 wt% NVP-PQ/PMMA, (c) is 0.10 wt% NVP-PQ/PMMA, (d) is 0.15 wt% NVP-PQ/PMMA and (e) is 0.20 wt% NVP-PQ/PMMA; Figure S2: DTG results of different concentrations of NVP-PMMA; Figure S3: Sample exposure area.
