# Supplementary material for: Impact of NVP Doping on the Holographic Properties of PQ/PMMA Holographic Storage Materials
Source: Polymers (Basel). 2025 Aug 27;17(17):2321. doi: 10.3390/polym17172321 (PMC12430988; doi:10.3390/polym17172321)
Supplement: Supplementary file 1 [file polymers-17-02321-s001.zip › polymers-3806790-supplementary.pdf]

Supporting Information

Impact of NVP Doping on the Holographic Properties of PQ/PMMA Holographic Storage Materials

Lin Peng[1], Junhui Wu[1], Shujun Zheng[1], Hongjie Liu[1], Ruying Xiong[1], Xueyan Chen[1], Xu Zheng[1], Xiao Lin[2,*], Xiaodi Tan [2,*]

1 College of Photonic and Electronic Engineering, Fujian Normal University, Fuzhou 350117, China

2 Information Photonics Research Center, Key Laboratory of Optoelectronic Science and for Medicine of Ministry of Education, Fujian Provincial Key Laboratory of Photonics Technology, Fujian Provincial Engineering Technology Research Center of Photoelectric Sensing Application, Fujian Normal University, Fuzhou 350117, China

* Email: xiaolin@fjnu.edu.cn; Email: xtan@fjnu.edu.cn

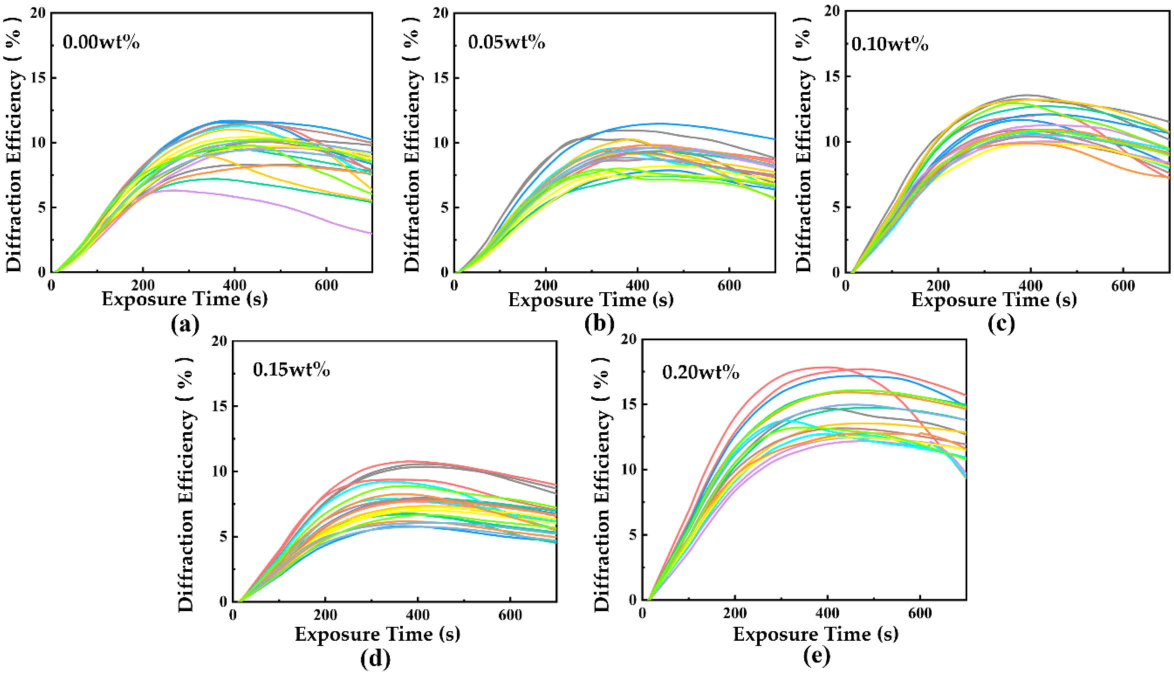

Figure S1. The diffraction efficiencies at multiple points on the same NVP-PQ/PMMA films with different doping concentrations, where (a) is 0.00wt% NVP-PQ/PMMA, (b) is 0.05wt% NVP-PQ/PMMA, (c) is 0.10wt% NVP-PQ/PMMA, (d) is 0.15wt% NVP-PQ/PMMA and (e) is 0.20wt% NVP-PQ/PMMA.

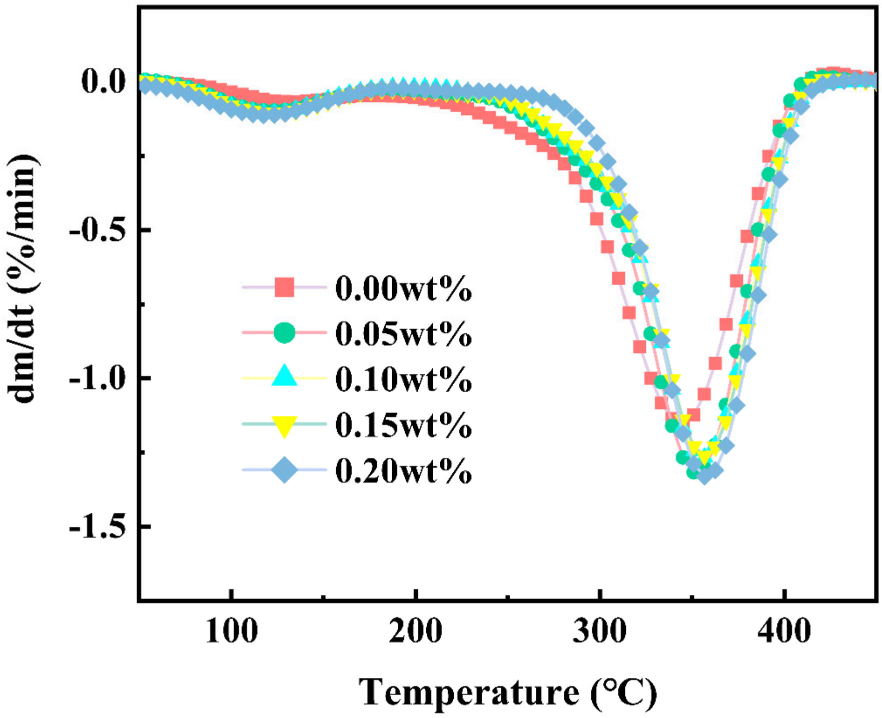

Figure S2. DTG results of different concentration of NVP-PMMA.

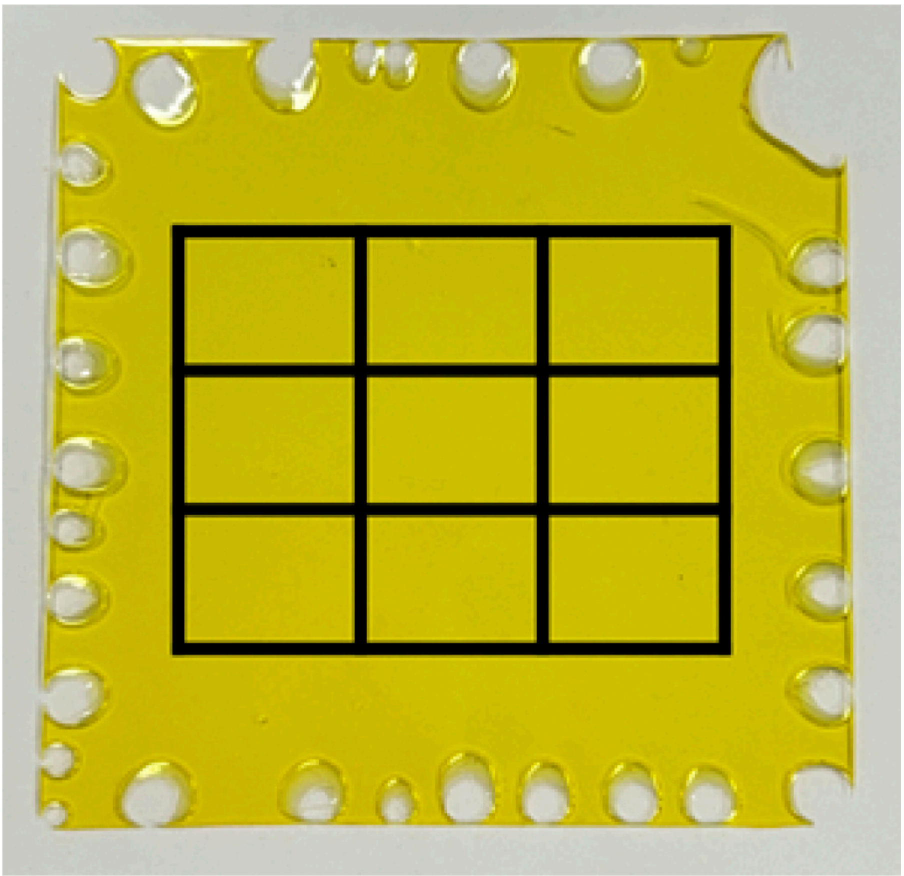

Figure S3. Sample exposure area
